# Physical Activity Promotion for Apprentices in Nursing Care and Automotive Mechatronics–Competence Counts More than Volume

**DOI:** 10.3390/ijerph17030793

**Published:** 2020-01-28

**Authors:** Johannes Carl, Eva Grüne, Johanna Popp, Klaus Pfeifer

**Affiliations:** Department of Sport Science and Sport, Friedrich Alexander-University Erlangen-Nürnberg, 91058 Erlangen, Germanyklaus.pfeifer@fau.de (K.P.)

**Keywords:** physical activity, health competence, apprentices, work ability, health literacy

## Abstract

Apprentices in the area of nursing care and automotive mechatronics are exposed to increased health risks. In this context, the promotion of physical activity (PA) is considered an effective strategy for the assurance of work ability. The goal of the PArC-AVE study was therefore to better understand the role of PA for apprentices employed in these two sectors. In an exploratory study, 55 apprentices wore an ActiGraph accelerometer over seven consecutive days and were subject to activity analysis. The objective accelerometer data (18,979 ± 3780 steps/day; 471.00 ± 159.75 min of moderate-to-vigorous PA/week), complemented by questionnaire data, indicated that most met the volume-based PA recommendations. Subsequently, we conducted a multicenter study comprising 745 apprentices from six vocational education institutions. Path analyses showed that competencies for health-enhancing PA were significantly related to indicators of work ability (0.180 ≤ *b* ≤ 0.452) and psychophysical health (0.139 ≤ *b* ≤ 0.347), whereas mere volume of PA was not (−0.048 ≤ *b* ≤ 0.080). In summary, apprentices of nursing care and automotive mechatronics showed high levels of PA. However, the results highlight the importance of competencies for health-enhancing PA. The PAHCO model could provide a useful framework for the conceptualization of effective interventions.

## 1. Introduction

The world of work is currently undergoing considerable transitions driven by phenomena such as digitization, technologization, or globalization. In addition, social shiftings such as demographic change present the world of work with additional challenges including ageing workforces or the shortage of skilled workers. As a result, not only occupational profiles and tasks are changing, but the demands placed on employees are as well. Flexibility and overarching competences are needed to adapt one’s own vocational qualifications to the changing needs of the labor market. To maintain employability in a changing world of work, lifelong learning plays a key role [1,2].

For a large number of young people who do not follow higher education after graduating from school, vocational education and training mark the first step into working life. Despite their recent entry into the labor market, young workers are already exposed to increased health risks and vulnerable to work-related diseases [3]. Studies have shown that the health burdens of apprentices are particularly high in occupations with high physical demands, such as the automotive industry and nursing care. For example, Betz and colleagues [4] found high prevalence rates for health problems such as back pain (58%) and headaches (45%) in a large-scale study with more than 3500 apprentices from the automotive industry. In terms of nursing care, a survey of 1119 students revealed comparable results, with around 50% of apprentices suffering from back pain or headaches at least once a week [5]. At the same time, studies indicated that the physical activity (PA) behavior of apprentices can be described as inadequate. A study by Bonevski and colleagues [6] showed that only 12% of vocational education and training students met the recommended PA guidelines of 150 min of moderate-to-vigorous PA per week. Two other studies reported that 42% of automotive mechatronics students [4] have a low PA level and 29% of nursing students [7] are physically active less than once a week.

As PA has the potential to make a substantial contribution to individuals’ health [8,9], PA-promoting measures as part of workplace health promotion have come to the forefront of science and have accumulated a growing amount of evidence [10]. The promotion of PA is not only seen as an effective strategy for preventing diseases, but also to ensure work ability [11]. In the field of vocational education and training, PA-promoting interventions are lacking [12]. Against this background, the PArC-AVE project (physical activity-related health competence in apprenticeship and vocational education), embedded in in the research consortium of Capital4Health, addressed the issue of PA promotion in vocational education and training in the sectors of automotive mechatronics and nursing care in Bavaria (in the south of Germany). The primary aim of the project was to develop and implement PA promoting measures tailored to the needs of the target group and the given setting through a co-creation approach involving members of the target group and other relevant actors from research, policy, and practice domains. Therefore, the goal of the present article was to characterize the PA behavior of the target group (Study A) and to investigate the role of PA and physical activity-related health competence (PAHCO) in the development of measures (Study B) using two cross-sectional studies with apprentices in nursing care and automotive mechatronics.

## 2. Study A

### 2.1. Background

In general, little is currently known about the PA behavior of apprentices. Although two single investigations have indicated that the PA behavior of the target group can be described as insufficient, results to-date have only been based on self-reported studies [4,7]. As this methodology is subject to the risk of over- and under-reporting, it is important to take a closer look at the PA behavior of this target population using both subjective and objective assessments (such as accelerometry). Especially in co-creation approaches, which is a promising strategy for the development of health-promoting measures tailored to environmental circumstances and the needs of the target group [13], it is necessary to acquire a better understanding of the PA behavior of the target group.

### 2.2. Methodology

#### 2.2.1. Participants and Measurement

Within the scope of the PArC-AVE Study, we contacted two vocational education centers in Ingolstadt (Bavaria, Germany) to inquire about participation in this study. For the real-life assessment of the PA behavior, we used the ActiGraph wGT3X (Pensacola, Florida) which has shown to be a valid and reliable tool for adults [14,15]. The 81 apprentices who gave informed consent to participate, were asked to wear the device on the right hip for seven consecutive days and to register the wearing time in a standardized logbook. The participants were advised to only put down the device during sleeping periods and water-based activities (e.g., swimming, showering). In accordance with recommendations for accelerometer studies, we chose a sampling frequency of 100 Hz and an epoch length of 15 s [16]. A single measurement day was considered valid if the participants wore the ActiGraph at least eight hours per day. A participant, in turn, was only included in the final analyses if at least five out of seven days turned out to be valid. This procedure led to a final sample size of *N* = 55. A total of 28 apprentices came from the nursing care sector (24 female, 4 male; age: 22.18 ± 4.10 years), while 27 apprentices came from the automotive mechatronics sector (23 male, 4 female; age: 18.26 ± 1.13 years).

To exclude potential single method bias and underpin the activity results with more evidence, the 55 apprentices also filled out the validated BSA Questionnaire [17]. The participants were instructed to report the frequency and duration of their activities executed in the past four weeks. This instrument differentiates between leisure-time/transportation activities (eight dimensions) on the one hand and sport-/exercise-related activities (up to three free specifications) on the other. Following an inclusive definition of PA, we relied on the overall index. To avoid over-reporting and outlier problems, we applied the winsorization technique, which cuts down any data points above the 95th percentile.

#### 2.2.2. Data Analysis

Accelerometer raw data was analyzed with the software ActiLife and the cut-point model by Freedson, Melanson, and Sirard [18] which assigns activity counts to MET values (Table 1). This transformation enables researchers to determine the time that participants spend in different intensity categories, i.e., sedentary behavior, light PA, and moderate-to-vigorous PA (MVPA). Non-wear time was defined by an interval of at least consecutive 60 min of zero counts while tolerating two minutes with a maximum 100 counts [19]. To avoid overestimation of steps, we applied a normal filter with adapted weights instead of using a low-frequency extension [20].

Priority was given to statistics describing the values across the different intensity areas. Differences between both sectors were explored using multivariate analysis of variance (MANOVA). Because apprentices from automotive mechatronics industry wore the accelerometer for a longer duration, we additionally computed a model with wearing time as a covariate (MANCOVA). The statistical analyses were undertaken with the software SPSS, Version 25 (IBM, Armonk, USA).

#### 2.2.3. Informed Consent and Ethics

All subjects gave their informed consent for inclusion before they participated in the study. The study was conducted in accordance with the Declaration of Helsinki, and the project was approved by the Ethics Committee of the University Erlangen-Nürnberg (May 11, 2015; Sign 128_15 Bc).

### 2.3. Results

The analyses (Table 1) revealed that the apprentices accumulated 372.99 ± 58.10 min of sedentary behavior and 253.93 ± 42.76 min of light PA per day. Moreover, the participants spent a total of 494.2 ± 174.2 min per week with PA in the moderate-to-vigorous intensity domain. On average, the apprentices from both sectors performed 18,979 ± 3780 steps per day. Nevertheless, there was a remarkable heterogeneity within the sample, with mean values ranging between 11,226 and 31,560 daily steps. Most apprentices seemed to be able to meet the recommendation to spend at least 150 min per week with MVPA [21]. Given the recorded range between 169.9 and 1124.4 min of MVPA per week, the percentage of individuals adhering to these guidelines was 100%. According to step-based guidelines, individuals should achieve more than 10,000 (adolescents aged 12–19) or 8000 (adults aged 20–65) steps per day [22]. In this regard, also all participating apprentices (100%) were able to meet this recommendation. 

In summary, the questionnaire data were in line with the accelerometer findings. In the area of nursing care, 25 of the 28 participants (89.3%) spent more than 150 min per week with physical activities. In the automotive mechatronics sector, 26 of 27 apprentices (96.3%) met this guideline [21]. On a descriptive basis, apprentices from the automotive mechatronics sector (512.1 ± 324.3 min/week) tended to report higher values of PA than apprentices from the nursing care sector (421.0 ± 290.5 min/week). However, the statistical group comparison was non-significant, *F* (1, 53) = 1.21, *p* > 0.05, ∆t = 91 min. 

### 2.4. Discussion

Even though the analyses demonstrated huge variations in the amount of PA among apprentices, it can be summarized that most participants in our study were able to achieve the volume-based PA recommendations for MVPA and the number of steps [21,22]. The values for MVPA have even been exceeded by a factor of more than three. Despite these tendencies, the results have to be treated with a certain caution. As one limitation, the inclusion criteria for wearing time have narrowed the sample size. This not only affected the power of this study, which is typical in the context of accelerometry [16], but may have also led to a certain selection bias [23] favoring the inclusion of people with a stronger interest in topics related to exercise, sport, and PA. The representativeness of the findings is also limited by the fact that the participants were recruited in two vocational schools located in one city. Moreover, it cannot be ruled out that the participants, consciously or unconsciously, may have increased their PA behavior as a result of being recorded [24]. In addition, hip-located accelerometry usually fails to sufficiently capture actions that dominantly concern the upper body such as manipulative tasks or strength training for upper extremity [25,26], activities that play an important role in nursing care and automotive mechatronics. Related to this problem, the methodology selected did not allow us to have a more differentiated look on the PA behavior (e.g., by attributing PA levels to concrete tasks or distinguishing between activities inside and outside of school). 

Despite the exploratory character of this study, we concluded that apprentices from the fields of nursing care and automotive mechatronics accumulate a considerable amount of physical activities over the course of a day. The findings point in the same direction as a recent study that included hospital workers [27], a population very similar to nursing apprentices. The registered values are outstanding in comparison to representative studies of the general population or adolescents of the same age [19] or studies examining other working populations that also used the ActiGraph (e.g., [28]). Accordingly, our objective and subjective data did not confirm self-report studies that highlight high prevalence of physical inactivity found in other or related sectors of vocational education [4,6,7]. We cannot rule out that the different values may partly result from methodological differences between the studies. For example, Lehmann and colleagues [7] only included activities that lasted at least 20 min. This may have underestimated the overall levels of PA. However, some studies undertook direct comparisons between different sectors, thus providing a more substantial view on potential PA level differences across fields of vocational education. For example, apprentices in the craft and industry sector displayed higher activity levels than those in service and trade sectors and those with a predominantly sedentary job [29]. Importantly, nursing apprentices in hospitals tend to be more engaged with PA than apprentices in early childhood education [30]. 

Against the backdrop of our findings, the focus should be turned to the physical demands that apprentices are facing in the specific areas of nursing care and automotive mechatronics during the workday. The practical work constitutes a large part of the vocational education program in these two sectors [31]. In the area of automotive mechatronics, for instance, apprentices are involved in vehicle production, repair, and maintenance. These occupational activities are usually characterized by walking around, retrieving tools from containers, and working on and in cars while frequently changing one’s body position. In this context, the role of specific working positions such as “overhead” work has been highlighted due to its considerable risk of physical impairment [32,33]. Likewise, nurses are required to walk around in order to move medical equipment and care for patients through handling or transferring them, so that they have to face intensive physical tasks within their job [27,34].

From a health promotion perspective, it can therefore be concluded that simply increasing the apprentices’ volume of PA (even more) is probably not an effective strategy for promoting the health of this target group. The so-called PA paradox assumes that the amount of PA within the occupational context is even likely to exert a detrimental influence on workers’ health [35,36,37,38]. A recent article discussed that too low intensities, too long activity durations, insufficient recovery times, inadequate postures, and a low activity control may lead to this negative effect [39]. Given these scholarly developments and results from our accelerometer study, it seems worth addressing individual’s coping mechanisms and resources in dealing with physical demands.

## 3. Study B

### 3.1. Background

The previous study revealed that the mere volume of PA does not seem to be an issue in apprentices of nursing care and automotive mechatronics. Supported by insights of the PA paradox, we instead deduced that focusing on individual’s coping mechanisms and resources in dealing with physical demands could present a more promising health-promoting strategy with this population. The goal of the next part of the PArC-AVE Study was to empirically test this assumption on a larger scale.

**Figure 1:**
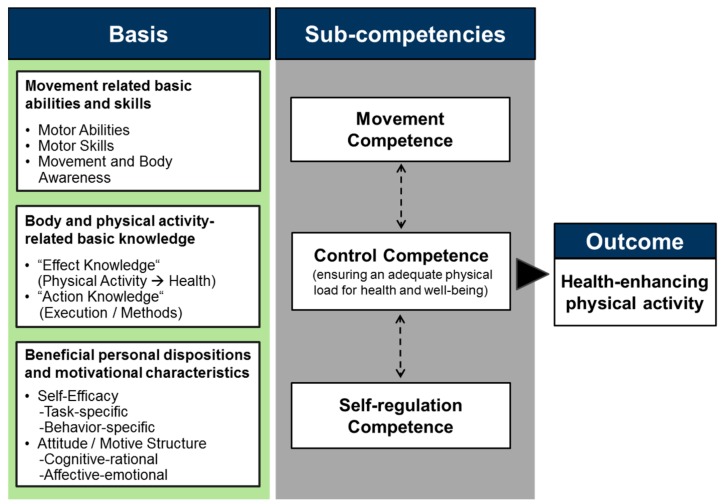
The model of physical activity-related health competence (PAHCO) [40].

As a suggested framework that specifies competencies when individuals have to master physical activities in a healthy manner, we used the model of physical activity-related health competence (PAHCO) [40,41]. Compared with the physical literacy concept, the PAHCO approach highlights the more functional role of these competencies being explicitly geared towards individual’s health [42]. A main characteristic of this framework (Figure 1) is its integrative core comprising person-related factors for ensuring both the quantity (volume of PA) and, importantly, the quality (health-relatedness) of physical activities. Specifically, the model postulates that individuals require three central sub-competencies: movement competence, self-regulation competence, and control competence. Movement competence refers to the direct motor requirements for mastering activities of daily life and participating in physical exercises. Self-regulation competence bundles the psychological factors ensuring a regular and self-determined execution of physical activities and exercises. Finally, as a more qualitative dimension, control competence guarantees that activities are not only performed as frequently and intensively as possible but that they are adequately aligned to the individual’s physical condition and psychological well-being as well. 

For workplace-based health promotion, the employees’ work ability and health are crucial outcomes for evaluating the success of interventions in this field [11,43]. Work ability can be defined as “an employee’s physical, psychological, and social capacity to work” [11]; its biopsychosocial understanding explains why researchers identified conceptual overlaps with the construct of health [11,44]. In general, having a good work ability and health is of major interest to almost all actors in the occupational setting, often providing the opportunity for “a win-win situation” [45]. On the one hand, it is possible to take an economic-utilitarian view on work ability and health, emphasizing the perspective of employers. For instance, studies have shown that positive values are indicative of a large amount of outcomes beneficial to the enterprise such as job performance, productivity [46,47,48], few sick days [49], and reduced risk of serious work disability [50]. On the other hand, the constructs can also be viewed from a personal growth or human resource standpoint. Through this lens, studies can be reported that demonstrate work ability and health are related to job satisfaction [51,52], lower turnover intentions [53], higher subjective well-being [54], and better quality of life [55]. Based on this assumption, companies should have an intrinsic interest in employing people who have a good work ability and health [56]. Due to their importance in the context of work, we drew on these two constructs as our primary outcomes.

Based on the insights from the first study and the evidence from the literature, we hypothesized that there was a small yet significant association between the apprentices’ amount of PA and their work ability [44] and health [8] levels. However, it was expected that aspects of PAHCO, which allow apprentices to better cope with physical and psychological work demands, were more strongly related to work ability and health than the mere PA volume. This would mean that the predictive contribution of the latter variable would be undermined in a combined analysis.

### 3.2. Methodology

#### 3.2.1. Participants and Measurement

To test this assumption, we developed a questionnaire study and asked six vocational education institutions in Bavaria (Germany) for their participation in this cross-sectional survey. Following their positive appraisal, the institutions were given the opportunity to decide whether they preferred a paper-pencil or an online questionnaire format. Data collection involving 768 participants occurred between July 2016 and January 2018. After data cleaning, a total of 496 apprentices from the automotive mechatronics sector (two institutions) and 249 apprentices from the nursing care sector (four institutions) remained in the final analyses. More information on the sample can be taken from Table 2.

**Figure A1:**
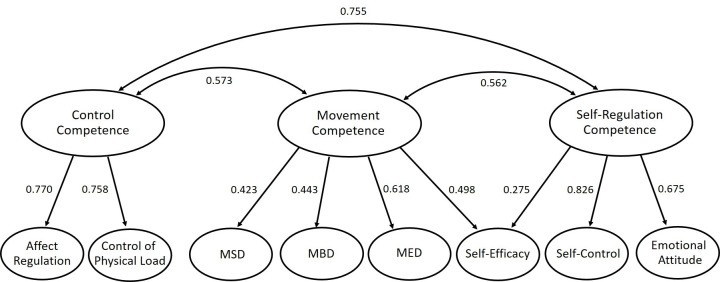
The second-order structure on PAHCO with the eight factors among apprentices.Note: MBA = manageability of balance demands, MSD = manageability of strength demands, MED = manageability of endurance demands.

The first outcome, work ability, was assessed via a short version of the German Work Ability Index (WAI-r [57]). This tool contains five sub-dimensions and eight items overall. The second outcome of psychophysical health was operationalized by the German version of the SF-12 Questionnaire which has been extensively tested for reliability and validity [58]. The instrument provides separate sum scores for physical and psychosocial health. In the present analysis, we totaled the two scores using a *z*-transformation algorithm. For the volume of physical activity, we again employed the BSA questionnaire [17]. Details to the instrument and its handling can be found in the methodology section of Study A. The questionnaire on physical activity-related health competence contained 34 items and eight subfactors. In this version, the instrument had already successfully been tested for reliability as well as factorial, discriminant, and criterion validity in apprentices of these two sectors [59,60]. The eight subfactors represent aspects of the three sub-competencies of movement competence, control competence, and self-regulation competence. In accordance with theoretical elaborations [40,41], the factors “affect regulation” and “control of physical load” can be interpreted as covering the sub-competence of control competence; the factors of “manageability of endurance demands” (MED), “manageability of strength demands” (MSD), “manageability of balance demands” (MBD), and “self-efficacy” can be considered aspects of movement competence; and finally, “emotional attitude towards PA”, “self-control”, and “self-efficacy” can be viewed as reflecting self-regulation competence. The present data set supports this assignment pattern (Figure A1) by exhibiting an “acceptable” [61] model fit, SB-χ²/df = 2.92, CFI = 0.926, RMSEA = 0.051 [CI_90_ = 0.048–0.054], SRMR = 0.060.

#### 3.2.2. Data Analysis

**Figure 2:**
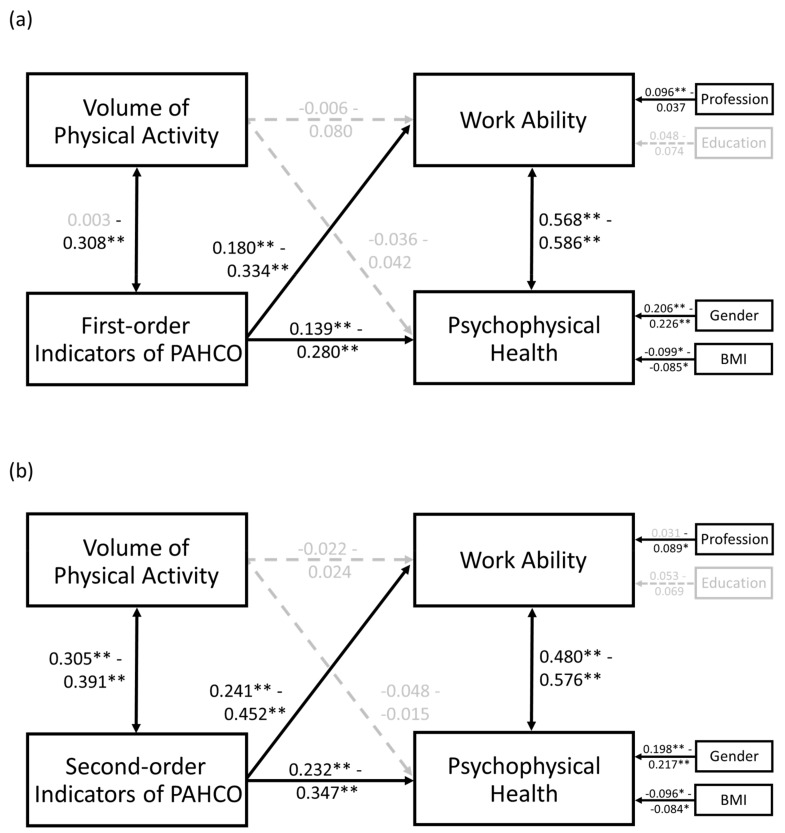
Path analysis with the manifest variables, covariates, correlations, and path coefficients. (**a**) Results with inclusion of the first-order indictors of PAHCO, (**b**) Results with inclusion of the second-order factors of PAHCO. Note: The significant variables, paths, and coefficients are displayed in black, the non-significant are displayed in grey. ** *p* < 0.00625, * *p* < 0.05 (only for covariates).

For testing our main hypothesis, we calculated a series of path models with four manifest variables (Figure 2). The scores of work ability as well as psychophysical health were treated as (correlated) dependent variables, while the volume of PA and a further PAHCO indicator (see below) served as (correlated) independent variables. 

In addition, we wanted to exclude the fact that the effects on the dependent variables resulted from the influence of relevant sociodemographic characteristics. Prior to main analysis, we therefore used linear regression modeling to screen the relevance of the following variables [44,62]: height-adjusted body weight (body mass index (BMI) in kg/m²), age (in years), level of education (four gradations), gender (dummy coded: female vs. male), and profession (dummy coded: nursing care vs. automotive mechatronics). The significant predictors from this initial analysis were added as control variables.

In all, we conducted eleven separate path analyses. Eight models contained the first-order sum scores of PAHCO (i.e., MSD, MED, MBD, control of physical load, affect regulation, self-control, emotional attitude toward PA, and self-efficacy). Specific information on items and psychometric characteristics can be retrieved from another study [59]. In addition, we computed three models comprising the second-order sum scores of movement competence, control competence, and self-regulation competence, all conceptualized as composites of a combination of first-order factors (Figure A1). The correlations and coefficients were freely estimated. The knowledge of reliabilities on the PAHCO scores allowed us to apply a correction of attenuation.

Due to the number of PAHCO indicators (*n* = 8), we anticipated multiple testing to be a serious problem [63]. We therefore applied Bonferroni correction with a more conservative critical significance level of *p* < 0.00625. Covariates were already considered when *p* < 0.05. Effect sizes were interpreted by following the suggestions of Cohen [64]: small effect *b* ≈ 0.10, moderate effect *b* ≈ 0.30, and strong effect *b* ≈ 0.50. We applied full information maximum likelihood (FIML) imputation techniques to counteract missing values. All analyses were performed with the R software, version 3.4.3, and the lavaan package [65].

### 3.3. Results

The initial covariate analysis (Table A1) revealed that apprentices from the automotive mechatronics sector (*β* = 0.130, *p* = 0.020) and those with a higher education degree (*β* = 0.098, *p* = 0.019) reported more favorable values of work ability. In addition, male individuals (*β* = 0.180, *p* < 0.001) and those with a lower BMI (*β* = −0.093, *p* = 0.016) showed better psychophysical health. Therefore, we consistently treated these variables as control variables.

In all path models, either with the first-order (Figure 2a) or second-order (Figure 2b) PAHCO indicators, the two outcomes of work ability and psychophysical health were strongly correlated with each other (0.480 ≤ *r* ≤ 0.586, *p* < 0.001). In accordance with a previous validation study [59], the PAHCO indicators were also significantly related to the volume of PA (0.200 ≤ *r* ≤ 0.391, *p* < 0.001), except of MBD (*b* = 0.003, *p* = 0.951). However, there was no linear relationship between the self-reported volume of PA and work ability (−0.022 ≤ *b* ≤ 0.080, *p* ≥ 0.059) and psychophysical health (−0.048 ≤ *b* ≤ 0.042, *p* ≥ 0.308).

The indicators of PAHCO, in contrast, were all significantly related to the two outcomes, even when considering the control variables. When analyzing the first-order paths to work ability outcome (Table 3), the coefficient was highest in magnitude for MSD (*b* = 0.334, *p* < 0.001) and emotional attitudes toward PA (*b* = 0.312, *p* < 0.001), whereas it was smallest for control of physical load (*b* = 0.188, *p* = 0.001) and MBD (*b* = 0.180, *p* = 0.001). The coefficients to the outcome of psychophysical health outcome were slightly lower, with emotional attitude toward PA (*b* = 0.280, *p* < 0.001) and self-control (*b* = 0.238, *p* < 0.001) showing the strongest associations, and self-efficacy (*b* = 0.150, *p* = 0.001) as well as MSD (*b* = 0.139, *p* = 0.001) displaying the weakest.

The sub-competencies, which embrace the idea of multiple skill and ability integration, also contributed significantly to the explanation of work ability (Table 4). We registered a small to moderate influence for control competence (*b* = 0.241, *p* < 0.001) and a moderate to strong influence for the measures of movement (*b* = 0.452, *p* < 0.001) and self-regulation competence (*b* = 0.373, *p* < 0.001). A similar pattern could be found for the outcome of psychophysical health (0.232 ≤ *b* ≤ 0.347, *p* < 0.001).

### 3.4. Discussion

The expected association of the PA volume with the outcome variables of work ability and health could not be recorded in this study. Our results run counter to the systematic review by Cadiz et al. [44] that consistently found small but positive relationships between PA and work ability. This difference, however, might be attributed to the fact that the nine studies included in the review concentrated mainly on more intensive forms of PA (exercise), other occupational groups, and, importantly, on much older employees. In older adults, the PA behavior can be interpreted as a more direct expression of the health status. Older individuals have probably more strongly profited from the positive health effects of lifetime-accumulated PA, causing stronger heterogeneity in older samples. An alternative explanation might be that apprentices of these two sectors amass too much activity over the course of their day, which is in line with findings from the first study. This constellation, especially when the percentage of occupational PA is high [35,37], could carry the middle-term risk of physical and mental overload. This could have impaired the work ability and health status of very physically active individuals. All these explanations might have shattered linear associations between the two outcomes and the volume of PA in apprentices of nursing care and automotive mechatronics. 

In accordance with our hypothesis, it could be shown that PAHCO indicators were substantially related to work ability and health when they were considered as predictors together with the volume of PA. More specifically, the path analyses revealed that the first-order factors on PAHCO had a varying effect on apprentices’ work ability and health. For instance, the competence to master endurance-related demands (MED) was similarly related to both outcomes (*∆b* = 0.032). The competence to master strength-related demands (MSD), in contrast, seemed to have a quite different impact (*∆b* = 0.195) on work ability (moderate effect) and health (small effect). The analyses demonstrated that self-control and emotional attitudes toward PA were the strongest psychological predictors across the models. Against the background of these detailed insights, it was worth breaking down the analysis to single competence elements. Ultimately, the condensed analysis with the second-order predictors of PAHCO highlighted the importance of all three competence areas. In accordance with the idea of multiple skill and ability integration, the aggregate scores had a higher predictive power than their corresponding sub-constructs.

Despite the evidence underlining the importance of physical activity-related health competencies in terms of apprentices’ work ability and health, we identified some limitations of this study. First, the cross-sectional character prevents us from drawing direct causal conclusions. For instance, we cannot completely exclude that impairments in apprentices’ work ability and health (e.g., caused by an illness or injury) may have impacted the PA behavior or the PAHCO assessment. Second, the selected PA instrument [17] did not allow us to distinguish between leisure-time and occupational PA levels. A differentiated look would have been beneficial in order to more strongly interpret the findings against the background of the PA paradox [36]. Third, we only involved apprentices from Germany, which, owing to the specificities of the vocational education system, compromised the cross-national generalizability of the findings. Fourth, our analysis would have been stronger if we had controlled for calorie intake and aspects of the work environment, such as regular overtime and shift work. Fifth, we exclusively relied on self-report data. For the concepts of work ability, psychophysical health, and PAHCO, there is, to our knowledge, no better alternative when striving for a multidimensional assessment. For the PA measurement, accelerometry would have been an option (see Study A). In such a case, however, it would have not been possible to achieve a comparable sample size, which is a strength of the present study.

## 4. Discussion

Study A enabled a quantification of the PA load that apprentices of the sectors nursing care and automotive mechatronics typically undergo throughout their day. The exceptional results induced us to focus on competencies favoring a health-oriented execution of physical activities and to investigate their role in the context of apprentices’ work ability and health. The theoretical background in the second study was provided by the PAHCO Model [40,41]. These competencies, conceptualized as latent dispositions, more strongly contributed to the explanation of work ability and health than the volume of apprentices’ PA behavior. The findings of both studies question the universal validity of the simple, quantitative formula “the more, the better“, which can be found in several recommendations and publications (for a similar criticism, see [66,67]). Instead, it may be beneficial to pay more attention to functional-qualitative aspects when conducting PA-related measures concerning this target group. For instance, the goals of conditioning and exercise, especially strength and endurance training, could be more closely matched with skills relevant to occupational tasks. Another potential implication to be drawn from the present study is that psychological and cognitive-reflective qualities in connection with physical demands should be fostered. Vocational education hardly addresses aspects dealing with ergonomics and physical demands [68]. The introduction of adequately designed education units might help apprentices increase their awareness of the role that an adequate execution of activities can play for their occupational life, physical health, and mental well-being. Ideally, teachers even incorporate psychological elements into their training components. In this context, it is possible to rely on evidence-based recommendations for developing effective interventions on specific PAHCO elements such as self-efficacy [69] or self-control [70].

In summary, the present study contributes to comprehending the role of PA for apprentices in nursing care and automotive mechatronics. Due to the severe shortage of skilled workers and the fact that apprentices are the “employees of tomorrow” [71], it is likely that several projects will follow, both in research and practice, that target apprentices of these two specific sectors. In this regard, the present study contributed to a better characterization of this target group. The second part of this article pointed to the importance of competencies for a healthy, physically active lifestyle. Several interventions already drew on PAHCO as a benchmark [72,73,74], which pleads for its use with apprentices as well. The PArC-AVE study, as one of these projects, combines the focus on PAHCO with a co-creation approach. Even though a qualitative study reported the implementation of cooperatively developed measures in settings of vocational education [75], it is not yet clear whether it was possible to foster aspects of PAHCO through such a complex approach. Evaluation studies are needed to prove the suitability of PAHCO for interventions.

## 5. Conclusions

The present article revealed that the largest part of apprentices from the sectors of nursing care and automotive mechatronics met the common PA guidelines in terms of the number of daily steps and time engaging in moderate-to-vigorous PA. In line with this finding, further analyses showed that physical activity-related health competencies which allow individuals to adequately cope with physical demands were more strongly associated with work ability and health than the mere volume of PA. The results of both studies suggest that workplace-based PA promotion for apprentices in these two sectors should focus on qualitative and competence-oriented dimensions of PA instead of simply following the quantitative formula “the more, the better”.

## Figures and Tables

**Table 1:** The different intensity categories and results of the accelerometer study.

Parameter	Activity counts [cpm] [18]	MET Values	Apprentices of nursing care (n = 28)	Apprentices of Automotive Mechatronics (n = 27)	Group Comparison	Group Comparison, Adjusted for Wearing Time
Sedentary Behavior (minutes /day)	0–99	<1.50	349.01 ± 54.81	397.86 ± 52.53	F(1, 53) = 11.4, *p* < 0.001 **, *d* = 0.91	F(1, 52) = 1.17, *p* = 0.284
Light PA (minutes/day)	100–1951	1.50–2.99	232.53 ± 37.09	276.13 ± 37.89	F(1, 53) = 18.6, *p* < 0.001 **, *d* = 1.16	F(1, 52) = 1.10, *p* = 0.300
Moderate PA (minutes/week)	1952–5724	3.00–5.99	415.64 ± 135.19	528.43 ± 168.16	F(1, 53) = 7.54, *p* < 0.001 **, *d* = 0.74	F(1, 52) = 0.027, *p* = 0.869
Vigorous PA (minutes/week)	≥5725	≥6.00	15.75 ± 17.32	30.83 ± 42.13	F(1, 53) = 3.05, *p* = 0.087	F(1, 52) = 0.798, *p* = 0.376
Number of Steps (per day)	-	-	16,822 ± 2,353	21,215 ± 3,704	F(1, 53) = 27.8, *p* < 0.001 **, *d* = 1.42	F(1, 52) = 2.12, *p* = 0.152
Wearing Time (minutes/day)	-	-	643.16 ± 47.80	753.88 ± 79.42	F(1, 53) = 39.6, *p* < 0.001 **, *d* = 1.70	-

Note: ** *p* < 0.01

**Table 2:** Description of the sample in Study B.

Variable	Description
Sample Size	*n* = 745
Profession	Automotive Mechatronics (66.6%), Nursing Care (33.4%)
Gender	Male (59.1%), Female (40.9%)
Age	19.06 ± 3.38 Years [Range 15–48]
Level of Education	Hauptschule/Middle School (22.3%), Realschule/Junior High School (57.3%), Fachhochschule/Senior High School (4.5%), Abitur/University Entrance Qualification (13.8%), Other Qualification (2.1%)
Body Mass Index [kg/m²]	23.10 ± 3.87 [Range 14.7–43.3]
Progress within the Apprenticeship Program	First Year (54.4%), Second Year (23.0%), Third Year (18.1%), Supplementary Year (4.5%)
Volume of Sport Activity (BSA) [minutes/week]	218.33 ± 256.50
Volume of Overall PA (BSA) [minutes/week]	451.41 ± 397.08
Work Ability (WAI-r) Index	30.14 ± 3.76 [Range 8–36]
Work Ability (WAI-r) Classification	Excellent (29.7%), Good (52.0%), Moderate (16.5%), Bad (1.8%)

**Table 3:** Results of the path analyses using the first-order indicators of PAHCO.

The Model and included variables		Outcome Work Ability			Outcome Psychophysical Health		
Model	Independent Variable	*b*	*z*	*p*	*b*	*z*	*p*
M1a	Volume of PA	0.031	0.748	0.455	0.017	0.423	0.672
	MSD	0.334	4.82	<0.001 **	0.139	3.31	0.001 **
M1b	Volume of PA	0.016	0.379	0.705	−0.014	−0.342	0.733
	MED	0.264	5.36	<0.001 **	0.232	5.18	<0.001 **
M1c	Volume of PA	0.080	1.89	0.059	0.042	1.02	0.308
	MBD	0.188	3.43	0.001 **	0.201	4.24	<0.001 **
M1d	Volume of PA	0.042	0.986	0.324	0.000	0.011	0.991
	Control of Physical Load	0.180	3.72	<0.001 **	0.189	4.05	<0.001 **
M1e	Volume of PA	0.032	0.756	0.450	−0.004	−0.100	0.921
	Affect Regulation	0.192	4.29	<0.001 **	0.170	4.02	<0.001 **
M1f	Volume of PA	0.016	0.397	0.691	−0.017	−0.414	0.679
	Self-Control	0.257	5.78	<0.001 **	0.238	5.70	<0.001 **
M1g	Volume of PA	−0.006	−0.146	0.884	−0.036	−0.869	0.385
	Emotional Attitude	0.312	7.81	<0.001 **	0.280	6.30	<0.001 **
M1h	Volume of PA	0.041	0.984	0.325	0.012	0.282	0.778
	Self-Efficacy	0.194	4.39	<0.001 **	0.150	3.20	0.001 **

Note: For reasons of presentation, covariates (profession and level of education for work ability; gender and BMI for psychophysical health) as well as correlations between the independent and dependent variables were not displayed. An attenuation correction was applied for relationships involving PAHCO as an indicator. ** *p* < 0.00625

**Table 4:** Results of the path analyses using the second-order indicators of PAHCO.

		Outcome Work Ability			Outcome Psychophysical Health		
Model	Independent Variable	*b*	*z*	*p*	*b*	*z*	*p*
M2a	Volume of PA	0.000	−0.006	0.995	−0.018	−0.450	0.653
	Movement Competence	0.452	6.87	<0.001 **	0.347	6.78	<0.001 **
M2b	Volume of PA	0.024	0.564	0.573	−0.015	−0.351	0.725
	Control Competence	0.241	4.42	<0.001 **	0.232	4.47	<0.001 **
M2c	Volume of PA	−0.022	−0.528	0.597	−0.048	−1.13	0.257
	Self-Regulation Competence	0.373	7.27	<0.001 **	0.328	6.36	<0.001 **

Note: For reasons of presentation, covariates (profession and level of education for work ability; gender and BMI for psychophysical health) as well as correlations between the independent and dependent variables were not displayed. An attenuation correction was applied for relationships involving PAHCO as an indicator. ** *p* < 0.00625.

## Appendix A

**Table A1:** Identification of potential confounders for work ability via multivariate regression modeling.

Covariate Model		Statistics of the Predictors		
Outcome Variable	Potential Covariate	*β*	*t*	*p*
Work Ability	Profession	0.130	2.33	0.020 *
	Age	−0.057	−1.38	0.168
	Level of Education	0.098	2.35	0.019 *
	Sex	−0.007	−0.133	0.894
	BMI	−0.004	0.106	0.916
Psychophysical Health	Profession	0.088	1.62	0.105
	Age	−0.045	−1.09	0.314
	Level of Education	0.044	1.07	0.287
	Sex	0.180	3.57	<0.001 **
	BMI	−0.093	−2.41	0.016 *

Note: Linear regression models for the identification of potential covariates, * *p* < 0.05. The variables profession (1 = nursing care, 2 = automotive mechatronics) and sex (1 = male, 2 = female) were dummy coded. ** *p* < 0.01, * *p* < 0.05

## References

[1] OECD (2019). OECD Employment Outlook 2019: The Future of Work.

[2] Federal Ministry of Education and Research (2016). Zukunft der Arbeit—Innovationen für die Arbeit von morgen. [The future of workInnovations for the work of tomorrow].

[3] Hanvold T.N., Kines P., Nykänen M., Thomée S., Holte K.A., Vuori J., Wærsted M., Veiersted K.B. (2019). Occupational safety and health among young workers in the Nordic countries: A systematic literature review. Saf. Health Work.

[4] Betz M., Graf-Weber G., Kapelke C., Wenchel K. (2012). Gesundheitsförderung in der überbetrieblichen Ausbildung am Beispiel des Kfz-Handwerks [Health promotion in intervocational education using the automotive trade as an example]. Dtsch med Wochenschr.

[5] Bomball J., Schwanke A., Stöver M., Görres S. (2010). Gesunde Pflege beginnt in der Pflegeausbildung [Healthy care begins with nursing training]. Die Schwester Der Pfleger.

[6] Bonevski B., Guillaumier A., Paul C., Walsh R. (2013). The vocational education setting for health promotion: A survey of students’ health risk behaviours and preferences for help. Health Promot. J. Austr..

[7] Lehmann F., von Lindeman K., Klewer J., Kugler J. (2014). BMI, physical inactivity, cigarette and alcohol consumption in female nursing students: A 5-year comparison. BMC Med. Educ..

[8] Warburton D.E.R., Bredin S.S.D. (2017). Health benefits of physical activity: A systematic review of current systematic reviews. Curr. Opin. Cardiol..

[9] Lee I.-M., Shiroma E.J., Lobelo F., Puska P., Blair S.N., Katzmarzyk P.T. (2012). Effect of physical inactivity on major non-communicable diseases worldwide: An analysis of burden of disease and life expectancy. Lancet.

[10] Proper K.I., van Oostrom S.H. (2019). The effectiveness of workplace health promotion interventions on physical and mental health outcomes—a systematic review of reviews. Scand. J. Work Environ. Health.

[11] Kuoppala J., Lamminpää A., Husman P. (2008). Work health promotion, job well-being, and sickness absences--a systematic review and meta-analysis. J. Occup. Environ. Med..

[12] Grüne E., Popp J., Carl J., Pfeifer K., Arampatzis A., Braun S., Schmitt K., Wolfarth B. (2019). Bewegungsförderungsmaßnahmen in der beruflichen Bildung: Ein systematisches Review [Physical activity interventions in vocational education and training: A systematic review]. Sport im öffentlichen Raum. Abstractband des 24. dvs-Hochschultags vom 18. bis 20. September 2019 in Berlin.

[13] Leask C.F., Sandlund M., Skelton D.A., Altenburg T.M., Cardon G., Chinapaw M.J.M., de Bourdeaudhuij I., Verloigne M., Chastin S.F.M. (2019). Framework, principles and recommendations for utilising participatory methodologies in the co-creation and evaluation of public health interventions. Res. Involv. Engagem..

[14] Lee J.A., Williams S.M., Brown D.D., Laurson K.R. (2015). Concurrent validation of the Actigraph gt3x+, Polar Active accelerometer, Omron HJ-720 and Yamax Digiwalker SW-701 pedometer step counts in lab-based and free-living settings. J. Sports Sci..

[15] Ozemek C., Kirschner M.M., Wilkerson B.S., Byun W., Kaminsky L.A. (2014). Intermonitor reliability of the GT3X+ accelerometer at hip, wrist and ankle sites during activities of daily living. Physiol. Meas..

[16] Migueles J.H., Cadenas-Sanchez C., Ekelund U., Delisle Nyström C., Mora-Gonzalez J., Löf M., Labayen I., Ruiz J.R., Ortega F.B. (2017). Accelerometer data collection and processing criteria to assess physical activity and other outcomes: A systematic review and practical considerations. Sports Med..

[17] Fuchs R., Klaperski S., Gerber M., Seelig H. (2015). Messung der Bewegungs- und Sportaktivität mit dem BSA-Fragebogen [Measurement of Physical Activity and Sport Activity With the BSA Questionnaire]. Zeitschrift für Gesundheitspsychologie.

[18] Freedson P.S., Melanson E., Sirard J. (1998). Calibration of the computer science and applications, Inc. accelerometer. Med. Sci. Sports Exerc..

[19] Troiano R.P., Berrigan D., Dodd K.W., Mâsse L.C., Tilert T., McDowell M. (2008). Physical activity in the United States measured by accelerometer. Med. Sci. Sports Exerc..

[20] Wanner M., Martin B.W., Meier F., Probst-Hensch N., Kriemler S. (2013). Effects of filter choice in GT3X accelerometer assessments of free-living activity. Med. Sci. Sports Exerc..

[21] WHO (2010). Global Recommendations on Physical Activity For Health.

[22] Tudor-Locke C., Craig C.L., Brown W.J., Clemes S.A., de Cocker K., Giles-Corti B., Hatano Y., Inoue S., Matsudo S.M., Mutrie N. (2011). How many steps/day are enough? For adults. Int. J. Behav. Nutr. Phys. Act..

[23] Weymar F., Braatz J., Guertler D., van den Berg N., Meyer C., John U., Felix S.B., Dörr M., Ulbricht S. (2015). Characteristics associated with non-participation in 7-day accelerometry. Prev. Med. Rep..

[24] Baumann S., Groß S., Voigt L., Ullrich A., Weymar F., Schwaneberg T., Dörr M., Meyer C., John U., Ulbricht S. (2018). Pitfalls in accelerometer-based measurement of physical activity: The presence of reactivity in an adult population. Scand. J. Med. Sci. Sports.

[25] Trost S.G., Zheng Y., Wong W.-K. (2014). Machine learning for activity recognition: Hip versus wrist data. Physiol. Meas..

[26] Swartz A.M., Strath S.J., Bassett D.R., O’Brien W.L., King G.A., Ainsworth B.E. (2000). Estimation of energy expenditure using CSA accelerometers at hip and wrist sites. Med. Sci. Sports Exerc..

[27] Jun S.Y., Kim J., Choi H., Kim J.S., Lim S.H., Sul B., Hong B.Y. (2019). Physical Activity of Workers in a Hospital. Int. J. Environ. Res. Public Health.

[28] Steeves J.A., Tudor-Locke C., Murphy R.A., King G.A., Fitzhugh E.C., Bassett D.R., van Domelen D., Schuna J.M., Harris T.B. (2018). Daily physical activity by occupational classification in us adults: NHANES 2005–2006. J. Phys. Act. Health.

[29] Kaminski A., Nauerth A., Pfefferle P.I. (2008). Gesundheitszustand und Gesundheitsverhalten von Auszubildenden im ersten Lehrjahr—Erste Ergebnisse einer Befragung in Bielefelder Berufskollegs [Health Status and Health Behaviour of Apprentices in the First Year of Apprenticeship—First Results of a Survey in Vocational Training Schools in Bielefeld]. Gesundheitswesen.

[30] Wirth T., Kozak A., Schedlbauer G., Nienhaus A. (2016). Health behaviour, health status and occupational prospects of apprentice nurses and kindergarten teachers in Germany: A cross-sectional study. J. Occup. Med. Toxicol..

[31] Deissinger T., Peterson P., Baker E., McGraw B. (2010). Dual System. International Encyclopedia of Education.

[32] Daub U., Budaker B., Schneider U., Bargende M., Reuss H.-C., Wiedemann J. (2015). Assistive technologies for workers in the automotive industry. 15. Internationales Stuttgarter Symposium.

[33] Vandergrift J.L., Gold J.E., Hanlon A., Punnett L. (2012). Physical and psychosocial ergonomic risk factors for low back pain in automobile manufacturing workers. Occup. Environ. Med..

[34] Lorusso A., Bruno S., L’Abbate N. (2007). A review of low back pain and musculoskeletal disorders among Italian nursing personnel. Ind. Health.

[35] Hall C., Heck J.E., Sandler D.P., Ritz B., Chen H., Krause N. (2019). Occupational and leisure-time physical activity differentially predict 6-year incidence of stroke and transient ischemic attack in women. Scand. J. Work Environ. Health.

[36] Hallman D.M., Jørgensen M.B., Holtermann A. (2017). On the health paradox of occupational and leisure-time physical activity using objective measurements: Effects on autonomic imbalance. PLoS ONE.

[37] Hu G.-C., Chien K.-L., Hsieh S.-F., Chen C.-Y., Tsai W.-H., Su T.-C. (2014). Occupational versus leisure-time physical activity in reducing cardiovascular risks and mortality among ethnic Chinese adults in Taiwan. Asia Pac. J. Public Health.

[38] Kulmala J., Ngandu T., Pajala S., Lehtisalo J., Levälahti E., Antikainen R., Laatikainen T., Oksa H., Peltonen M., Rauramaa R. (2016). Leisure-time and occupational physical activity in early and late adulthood in relation to later life physical functioning. J. Phys. Act. Health.

[39] Holtermann A., Krause N., van der Beek A.J., Straker L. (2018). The physical activity paradox: Six reasons why occupational physical activity (OPA) does not confer the cardiovascular health benefits that leisure time physical activity does. Br. J. Sports Med..

[40] Sudeck G., Pfeifer K. (2016). Physical activity-related health competence as an integrative objective in exercise therapy and health sports – conception and validation of a short questionnaire. German J. Exerc. Sport Res..

[41] Pfeifer K., Sudeck G., Geidl W., Tallner A. (2013). Bewegungsförderung und Sport in der Neurologie – Kompetenzorientierung und Nachhaltigkeit [Physical activity enhancement and sports in neurology –competence orientation and sustainability]. Neurol. Rehabil..

[42] Haible S., Volk C., Demetriou Y., Höner O., Thiel A., Sudeck G. (2020). Physical activity-related health competence, physical activity, and physical fitness: analysis of control competence for the self-directed exercise of adolescents. Int. J. Environ. Res. Public Health.

[43] Rongen A., Robroek S.J.W., van Lenthe F.J., Burdorf A. (2013). Workplace health promotion: A meta-analysis of effectiveness. Am. J. Prev. Med..

[44] Cadiz D.M., Brady G., Rineer J.R., Truxillo D.M., Wang M. (2019). A review and synthesis of the work ability literature. Work Aging Retire..

[45] Ilmarinen J. (2019). From work ability research to implementation. Int. J. Environ. Res. Public Health.

[46] Ford M.T., Cerasoli C.P., Higgins J.A., Decesare A.L. (2011). Relationships between psychological, physical, and behavioural health and work performance: A review and meta-analysis. Work Stress.

[47] Jansson I., Björklund A., Perseius K.-I., Gunnarsson A.B. (2015). The concept of ‘work ability’ from the view point of employers. Work J. Prev. Assesment Rehabil..

[48] Vänni K., Virtanen P., Luukkaala T., Nygård C.-H. (2012). Relationship between perceived work ability and productivity loss. Int. J. Occup. Saf. Ergon..

[49] Reeuwijk K.G., Robroek S.J.W., Niessen M.A.J., Kraaijenhagen R.A., Vergouwe Y., Burdorf A. (2015). The prognostic value of the work ability index for sickness absence among office workers. PLoS ONE.

[50] Alavinia S.M., de Boer A.G.E.M., van Duivenbooden J.C., Frings-Dresen M.H.W., Burdorf A. (2009). Determinants of work ability and its predictive value for disability. Occup. Med..

[51] Faragher E.B., Cass M., Cooper C.L. (2005). The relationship between job satisfaction and health: A meta-analysis. Occup. Environ. Med..

[52] Olsen E., Bjaalid G., Mikkelsen A. (2017). Work climate and the mediating role of workplace bullying related to job performance, job satisfaction, and work ability: A study among hospital nurses. J. Adv. Nurs..

[53] Camerino D., Conway P.M., van der Heijden B.I.J.M., Estryn-Béhar M., Costa G., Hasselhorn H.-M. (2008). Age-dependent relationships between work ability, thinking of quitting the job, and actual leaving among Italian nurses: A longitudinal study. Int. J. Nurs. Stud..

[54] Sjogren-Ronkä T., Ojanen M.T., Leskinen E.K., Mustalampi S.T., Mälkiä E.A. (2002). Physical and psychosocial prerequisites of functioning in relation to work ability and general subjective well-being among office workers. Scand. J. Work Environ. Health.

[55] Sörensen L.E., Pekkonen M.M., Männikkö K.H., Louhevaara V.A., Smolander J., Alén M.J. (2008). Associations between work ability, health-related quality of life, physical activity and fitness among middle-aged men. Appl. Ergon..

[56] Ilmarinen J. (2009). Work ability—a comprehensive concept for occupational health research and prevention. Scand. J. Work Environ. Health.

[57] Hetzel C., Baumann R., Bilhuber H., Mozdzanowski M. (2014). Ermittlung der Arbeitsfähigkeit anhand eines reduzierten Work Ability Index (WAI-r) [Determination of work ability by a Work Ability Index short form (WAI-r)]. ASU Arb. Soz. Umw..

[58] Morfeld M., Kirchberger I., Bullinger M. (2011). SF-36 Fragebogen zum Gesundheitszustand—DeutscheVersion des Short Form-36 Health Survey.

[59] Carl J., Geidl W., Sudeck G., Schultz K., Pfeifer K. (2020). Competencies for a healthy physically active lifestyle —Validation of an integrative model. ResearchSquare.

[60] Carl J., Semrau J., Pfeifer K., Murphy M., Boreham C., De Vito G., Tsolakidis E. (2018). Physical Activity-Related Health Competence: Using the PArC-AVE Study for an Extended Model Validation. Abstract Book of the 23rd Annual Congress of the Eurpean College of Sport Sciences, Dublin, Ireland, 4–7 July 2018.

[61] Backhaus K., Erichson B., Weiber R. (2015). Fortgeschrittene Multivariate Analysemethoden—Eine anwendungsorientierte Einführung [Advanced multivariate methods of analysis—An application-oriented introduction], 3, revised and actualized.

[62] El Fassi M., Bocquet V., Majery N., Lair M.L., Couffignal S., Mairiaux P. (2013). Work ability assessment in a worker population: Comparison and determinants of work ability index and work ability score. BMC Public Health.

[63] Streiner D.L., Norman G.R. (2011). Correction for multiple testing: Is there a resolution?. Chest.

[64] Cohen J. (1988). Statistical power analysis for the behavioral sciences.

[65] Rosseel Y. Package lavaan. https://cran.r-project.org/web/packages/lavaan/lavaan.pdf.

[66] Warburton D.E.R., Bredin S.S.D. (2016). Reflections on physical activity and health: what should we recommend?. Can. J. Cardiol..

[67] Warburton D.E.R., Taunton J., Bredin S.S.D., Isserow S.H. (2016). The risk-benefit paradox of exercise. BC Med J..

[68] Tamminen-Peter L., Östring E., Sormunen E., Cotrim T.P., Serranheira F., Suosa P., Hignett S., Albolino S., Tartaglia R. (2019). Improving ergonomics competences in the social and health care sector in Finland. Health and Social Care Systems of the Future: Demographic Changes, Digital Age and Human Factors.

[69] Ashford S., Edmunds J., French D.P. (2010). What is the best way to change self-efficacy to promote lifestyle and recreational physical activity? A systematic review with meta-analysis. Br. J. Health Psychol..

[70] Friese M., Hofmann W., Wiers R.W. (2011). On taming horses and strengthening riders: Recent developments in research on interventions to improve self-control in health behaviors. Self Identity.

[71] European Centre for the Development of Vocational Training Insights into skill shortaged and skill mismatch: Learning from Cedefop’s European skills and jobs survey. https://www.cedefop.europa.eu/files/3075_en.pdf.

[72] Bruland D., Voß M., Schulenkorf T., Latteck Ä.-D. (2019). Mit Schwung und Energie durch den Tag. Partizipative Forschung zur Förderung der bewegungsbezogenen Gesundheitskompetenz bei Menschen mit Lernschwierigkeiten [With enthusiasm and energy through the day. Participatory research to promote physical-activity-related health literacy in people with intellectual disabilities]. Prävention Gesundh..

[73] Haible S., Volk C., Demetriou Y., Höner O., Thiel A., Trautwein U., Sudeck G. (2019). Promotion of physical activity-related health competence in physical education: Study protocol for the GEKOS cluster randomized controlled trial. BMC Public Health.

[74] Geidl W., Semrau J., Streber R., Lehbert N., Wingart S., Tallner A., Wittmann M., Wagner R., Schultz K., Pfeifer K. (2017). Effects of a brief, pedometer-based behavioral intervention for individuals with COPD during inpatient pulmonary rehabilitation on 6-week and 6-month objectively measured physical activity: Study protocol for a randomized controlled trial. Trials.

[75] Popp J., Carl J., Grüne E., Semrau J., Gelius P., Pfeifer K. (2020). Physical activity promotion in German vocational education: Does capacity building work?. Health Promot. Int..

